# The M18 aspartyl aminopeptidase of *Plasmodium falciparum *binds to human erythrocyte spectrin *in vitro*

**DOI:** 10.1186/1475-2875-7-161

**Published:** 2008-08-22

**Authors:** Sonja B Lauterbach, Theresa L Coetzer

**Affiliations:** 1 Department of Molecular Medicine and Haematology, National Health Laboratory Service, School of Pathology, University of the Witwatersrand, Parktown, Johannesburg 2193, Republic of South Africa

## Abstract

**Background:**

During erythrocytic schizogony, *Plasmodium falciparum *interacts with the human erythrocyte membrane when it enters into, grows within and escapes from the erythrocyte. An interaction between the *P. falciparum *M18 aspartyl aminopeptidase (*Pf*M18AAP) and the human erythrocyte membrane protein spectrin was recently identified using phage display technology. In this study, recombinant (r) *Pf*M18AAP was characterized and the interaction between the enzyme and spectrin, as well as other erythrocyte membrane proteins, analyzed.

**Methods:**

r*Pf*M18AAP was produced as a hexahistidine-fusion protein in *Escherichia coli *and purified using magnetic bead technology. The pI of the enzyme was determined by two-dimensional gel electrophoresis and the number of subunits in the native enzyme was estimated from Ferguson plots. The enzymatic activity over a pH and temperature range was tested by a coupled enzyme assay. Blot overlays were performed to validate the spectrin-*Pf*M18AAP interaction, as well as identify additional interactions between the enzyme and other erythrocyte membrane proteins. Sequence analysis identified conserved amino acids that are expected to be involved in cofactor binding, substrate cleavage and quaternary structure stabilization.

**Results:**

r*Pf*M18AAP has a molecular weight of ~67 kDa and the enzyme separated as three entities with pI 6.6, 6.7 and 6.9. Non-denaturing gel electrophoresis indicated that r*Pf*M18AAP aggregated into oligomers. An *in vitro *coupled enzyme assay showed that r*Pf*M18AAP cleaved an N-terminal aspartate from a tripeptide substrate with maximum enzymatic activity at pH 7.5 and 37°C. The spectrin-binding region of *Pf*M18AAP is not found in *Homo sapiens, Saccharomyces cerevisiae *and other*Plasmodium *species homologues. Amino acids expected to be involved in cofactor binding, substrate cleavage and quaternary structure stabilization, are conserved. Blot overlays with r*Pf*M18AAP against spectrin and erythrocyte membrane proteins indicated that r*Pf*M18AAP binds to spectrin, as well as to protein 4.1, protein 4.2, actin and glyceraldehyde 3-phosphate dehydrogenase.

**Conclusion:**

Studies characterizing r*Pf*M18AAP showed that this enzyme interacts with erythrocyte spectrin and other membrane proteins. This suggests that, in addition to its proposed role in hemoglobin digestion, *Pf*M18AAP performs other functions in the erythrocyte host and can utilize several substrates, which highlights the multifunctional role of malaria enzymes.

## Background

Malaria, caused by an Apicomplexan of the genus *Plasmodium*, is one of the most prevalent and lethal diseases affecting the human race. Yearly 300–660 million people are diagnosed with clinical malaria [1] and the World Health Organization estimates that annually 2.7 million deaths are attributed to this disease [2]. The highest incidence of malaria, especially the severest form of malaria, caused by *Plasmodium falciparum*, occurs in Africa [1].

After transmission to humans, *P. falciparum *spends the disease-causing stage of its life cycle in erythrocytes. Entry into the erythrocyte, parasite growth and escape from the host cell all require protein-protein interactions between the parasite and the human erythrocyte membrane, which makes the study of these interactions a key issue in malaria research.

The erythrocyte membrane skeleton is located just beneath the erythrocyte membrane and consists of a two-dimensional network of spectrin tetramers linked to junctional complexes. Spectrin tetramers are the predominant proteins in the membrane skeleton and consist of two supercoiled rope-like spectrin-heterodimers which self-associate to form the tetramer. Each heterodimer contains an alpha and beta spectrin chain composed of multiple homologous 106 amino acid residue motifs (termed spectrin repeats) which fold into triple alpha-helical bundles [3]. Spectrin tetramers are long flexible molecules in the lattice framework that lend stability and flexibility to the membrane skeleton and are responsible for maintaining the shape of the erythron. Junctional complexes are located at the distal end of the spectrin tetramer, where the N-termini of six beta spectrin chains link to two actin protofilaments via protein 4.1 and dematin [3].

The malaria parasite initiates invasion by interacting with the glycophorins and band 3 that are exposed on the erythrocyte membrane surface. Several parasite ligands, for example, merozoite surface protein 1 (MSP-1) [4], MSP-9 [5], erythrocyte binding antigen 175 (EBA-175) [6] and EBA-140 [7] have been shown to interact with erythrocyte receptors, and some ligands, such as MSP-1, also interact with the spectrin molecules lying beneath the membrane [8]. Other parasite proteins, such as serine-rich antigen (SERA), the rhoptry proteins [9], ring-infected erythrocyte surface antigen (RESA), and gp76 [10] induce structural changes in the erythrocyte membrane phospholipids and the underlying membrane skeleton, which allow the parasite to enter the erythrocyte. Of these proteins, RESA has been shown to interact with spectrin by acting as a chaperone during the repair of the membrane skeleton after invasion has been completed. The protein binds to beta spectrin, close to the self-association site where it stabilizes the spectrin tetramer, thereby increasing the thermostability of the erythrocyte and preventing further invasion by other malaria parasites [11].

During parasite growth, striking structural and morphological changes are induced in the host cell. These include the loss of the typical erythrocyte shape, alterations in the mechanical properties of the cell, and modifications of the phosphorylation state of erythrocyte membrane skeleton proteins. Knobs, which play a role in sequestration, are also introduced on the host cell surface. Numerous parasite proteins, for example, *P. falciparum *erythrocyte membrane protein 3 (PfEMP-3), mature parasite-infected surface antigen (MESA), *falciparum*-exported serine/threonine kinase (FEST), and *falciparum *interspersed repeat antigen (FIRA), are associated with the membrane skeleton [12]. Knob-associated histidine-rich protein (KAHRP) and PfEMP-1 are present in the knobs, which interact with erythrocyte membrane skeleton proteins, such as spectrin, protein 4.1 and actin [12]. For example, MESA binds to protein 4.1 [13] and KAHRP interacts with repeat 4 on alpha spectrin [14], as well as the phosphorylated band 3-binding domain of ankyrin [15]. The cytoplasmic domain of PfEMP-1 also binds to the spectrin-actin junction of the membrane skeleton [16].

The release of the parasite from its host cell also depends on parasite-erythrocyte membrane interactions and several parasite enzymes have been suggested to play roles in the breakdown of the erythrocyte membrane skeleton. For example, a 37 kDa acidic protease cleaves beta spectrin and protein 4.1 [17]. Plasmepsin-II, which has a primary role in hemoglobin digestion, cleaves the SH3 domain of alpha spectrin at a neutral pH, and also interacts with actin and protein 4.1 [18]. Another food vacuole enzyme, the cysteine protease falcipain-2, cleaves ankyrin and protein 4.1 within the spectrin-actin-binding domain [19].

Previous work from our laboratory, which involved biopanning a *P. falciparum *phage-display library against erythrocyte spectrin, revealed that a 33 amino acid peptide of *Pf*M18AAP interacts with the erythrocyte membrane skeleton [20]. In this study the biochemical characteristics of the full length r*Pf*M18AAP were evaluated and the protein-protein interactions between r*Pf*M18AAP and spectrin, as well as other erythrocyte membrane proteins, were investigated.

## Methods

### Sequence analysis of *Pf*M18AAP

Aspartyl aminopeptidase proteases from *H. sapiens *(Q9ULAO), *S. cerevisiae *(P38821), *Plasmodium falciparum *(PFI1570c), *Plasmodium chabaudi chabaudi *(PC000238.00.0), *Plasmodium yoelii yoelii *(PY03205), *Plasmodium knowlesi *(PKH_073050), and *Plasmodium vivax *(Pv087090) were retrieved from the NCBI and PlasmoDB (version 5.3) database. The sequences were aligned using ClustalW at EMBL-EBI .

### Preparation of r*Pf*M18AAP

*Plasmodium falciparum *was cultured *in vitro *by the method of Trager and Jensen [21] and genomic DNA extracted by first lysing the erythrocytes and subsequently boiling the parasites to release the DNA [22]. The *Pf*M18AAP sequence (nucleotides 7–1689 of gene PFI1570c taken from the PlasmoDB (version 5.3) database) was amplified from the parasite DNA by PCR, using sequence specific primers containing 5' *Nde*I and 3'*BamH*I recognition sequences (restriction enzyme recognition sequence is in bold; F: 5' CTGAGGAACTG**CATATG**AAGAAAGCTAGGGAATACGCC 3'; R: 5' TGTGCT**GGATCC**TCATAAGACTTGGTTGATGTAGG 3'). The amplified DNA was inserted into the pET15-b vector (Novagen, USA), the insert sequenced and BL21-CodonPlus^® ^(DE3) RIL competent cells (Stratagene, USA) were transformed with the vector construct. r*Pf*M18AAP, containing an N-terminal hexahistidine-tag, was produced in Overnight Express™ Instant TB Medium (Novagen, USA) and purified from *E. coli *extracts using HIS-Select™ Magnetic Agarose Beads (Sigma, USA). The r*Pf*M18AAP concentration was determined spectrophotometrically at 595 nm using the Coomassie Plus^® ^Protein Assay Reagent Kit (Pierce, USA) and the purified protein sample analyzed by sodium dodecylsulphate 10% polyacrylamide gel electrophoresis (SDS-PAGE) [23].

### *Pf*M18AAP isoelectric point and native oligomeric state determination

The hexahistidine-tag was removed from r*Pf*M18AAP with the THROMBIN CleanCleave™ KIT (Sigma, USA) and the isoelectric point (pI) of r*Pf*M18AAP determined by two-dimensional gel electrophoresis [24] against 2-D SDS-PAGE Standards (Bio-Rad, USA). The first dimension isoelectric focusing (IEF) gel contained the Bio-Lyte^® ^3/10 Ampholyte (Bio-Rad, USA) and the second dimension Laemmli SDS-polyacrylamide gel [23] contained 13% acrylamide. The proteins were visualized by silver staining.

The number of subunits of native r*Pf*M18AAP was determined by Ferguson plots [25]. The protein was electrophoresed through non-denaturing agarose-acrylamide tube gels containing 3, 4, 5, 6, 7, 8, 9, and 10% acrylamide and 0.3% agarose [26]. The approximate molecular weight of each r*Pf*M18AAP subunit was determined from a standard curve constructed from the relative mobility of bovine serum albumin (BSA) (monomer = 66 kDa; dimer = 132 kDa) and human erythrocyte spectrin (dimer = 460 kDa; tetramer = 920 kDa; hexamer = 1380 kDa). Tube gels were cut in half to facilitate transfer onto Hybond™-C Extra Nitrocellulose membrane (Amersham, UK) and the r*Pf*M18AAP detected with 1:2000 Penta·His™ HRP Conjugate antibody (Qiagen, Germany) using the SuperSignal^® ^West Pico Chemiluminescent Substrate (Pierce, USA).

### r*Pf*M18AAP enzyme assay

A coupled enzyme assay using 0.3 mM Asp-Ala-Pro-beta-Napthylamide (Peptides International, USA) and 0.0025 U dipeptidyl peptidase IV (Sigma, USA) in 50 mM Tris-HCl (pH 7.5) was used to study the enzymatic activity of 2.5 μg r*Pf*M18AAP in a final volume of 50 μl [27]. To determine the optimum pH, the enzyme assay was performed with 50 mM Tris-HCl buffer at pH 6.8, 7.5, 8.0, 8.5 and 9.0, or 0.1 M sodium citrate buffer at pH 5.3, 6.0 and 6.5. A temperature study was completed in 50 mM Tris-HCl buffer (pH 7.5) at 25, 30, 33, 37 and 39°C.

### Blot overlay assays

Blot overlay assays were used to confirm the interaction between r*Pf*M18AAP and erythrocyte spectrin, as well as to study the protein-protein interactions between r*Pf*M18AAP and other erythrocyte membrane proteins.

Approximately 1 μg spectrin tetramers and dimers [28], 100 ng r*Pf*M18AAP (positive control) and 100 ng BSA (negative control) were applied to a Hybond™-C Extra Nitrocellulose membrane in a Bio-Rad Bio-Dot^® ^SF chamber (Bio-Rad, USA). To test the interaction of r*Pf*M18AAP with other erythrocyte membrane proteins, 100 ng r*Pf*M18AAP (positive control), 100 ng BSA (negative control) and 20 μg erythrocyte membrane proteins [28] were electrophoresed through a Laemmli SDS 9% polyacrylamide gel [23] or a Fairbanks SDS 3.5–17.5% polyacrylamide gel [29,30] and transferred onto a Hybond™-C Extra Nitrocellulose membrane. Membranes were blocked with 5% BSA in TBS (10 mM Tris-HCl, 150 mM NaCl, pH 7.5) and subsequently overlaid for 1 hr at room temperature with 1.25–2.5 μg r*Pf*M18AAP in either 50 mM Tris-HCl (pH 7.5), or 50 mM Tris-HCl (pH 7.5) containing 150 mM NaCl. Membranes were washed four times for 10 minutes in wash buffer (20 mM Tris-HCl, 500 mM NaCl, 0.12% Tween^®^-20 (v/v), 0.2% Triton^® ^X-100 (v/v), pH 7.5) and once for 10 minutes in TBS. The membranes were fixed for 20 minutes with 0.5% (v/v) formaldehyde followed by a 10 minute wash with TBS. The presence of r*Pf*M18AAP was detected with 1:2000 Penta·His™ HRP Conjugate antibody using the SuperSignal^® ^West Pico Chemiluminescent Substrate.

## Results and Discussion

### Analysis of the *Pf*M18AAP protein sequence and spectrin-binding region

*Pf*M18AAP is classified as an M18 aminopeptidase in the MEROPS database [31]. The 33 amino acid spectrin-binding region of *Pf*M18AAP, which was initially identified by phage display [20], was not found in the *H. sapiens *or *S. cerevisiae *sequences (Additional File 1). This region may represent a special evolutionary adaptation of *P. falciparum*, since it is specific for *Pf*M18AAP and is not found in other *Plasmodium *species. Despite this species-specific spectrin-binding insert, there is a 64–74% sequence identity between *Pf*M18AAP and the *Plasmodium *homologues. The sequence probably forms a loop on the surface of the active enzyme [32] and could, therefore, allow *Pf*M18AAP to bind to spectrin, without interfering with the enzymatic function.

*Pf*M18AAP is a metalloprotease and requires cofactors, probably cobalt [32], for enzymatic activity. These cofactors are bound by specific amino acids that are present in all the M18 aminopeptidases (Additional File 1). Conserved amino acids are labeled according to the amino acid numbers of the human aspartyl aminopeptidase [33] and the amino acid numbers of *Pf*M18AAP are given in brackets. The five amino acids, H94, D264, E302, D346 and H440 (H86, D324, E380, D434 and H534) involved in cofactor binding [31], as well as the two amino acids, D96 and E301 (D88 and E379) involved in substrate cleavage [31] are conserved in *Pf*M18AAP. Two additional histidines, H170 (H160), involved in enzymatic activity [33] and H352 (H440), involved in quaternary structure stabilization [33], are also present in all the sequences (Additional File 1).

### Biochemical characterization of r*Pf*M18AAP

**Figure 1:**
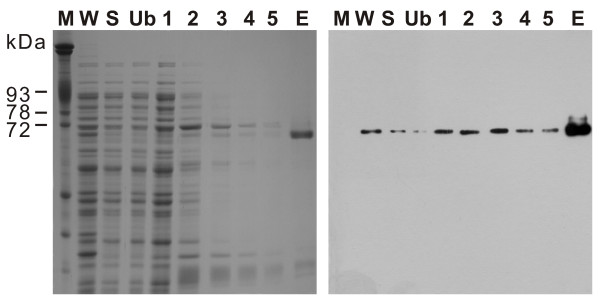
**Affinity purification of r*Pf*M18AAP**. SDS-polyacrylamide gel (left) and immunoblot (right) showing the purification of r*Pf*M18AAP. Lane M – erythrocyte membrane proteins; W – induced *E. coli *whole cell extract; S – soluble protein fraction after cell lysis; Ub – protein fraction that did not bind to the HIS-Select™ Magnetic Agarose Beads; 1–5 – consecutive 20 mM imidazole washes; E – 200 mM imidazole elution of r*Pf*M18AAP.

**Figure 2:**
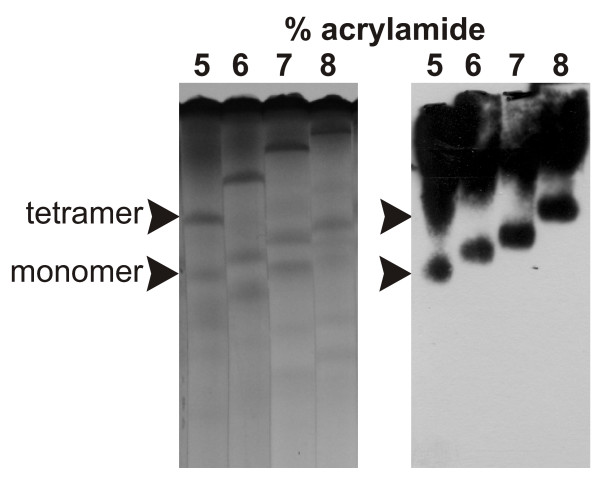
**Non-denaturing electrophoresis of r*Pf*M18AAP**. Non-denaturing agarose/polyacrylamide gels (left) and immunoblot (right) showing the monomer and tetramer of r*Pf*M18AAP. The protein was electrophoresed through non-denaturing gels containing 5, 6, 7, and 8% acrylamide. r*Pf*M18AAP was detected with the PentaHis™ HRP Conjugate antibody.

Only a small proportion of r*Pf*M18AAP was expressed as soluble protein after induction in Overnight Express™ Instant TB Medium and therefore only ~1 μg of soluble protein was obtained from a one liter *E. coli *culture. The N-terminal hexahistidine-tag present in r*Pf*M18AAP was detected by Western blot analysis (Figure 1) and purification of r*Pf*M18AAP from *E. coli *extracts yielded a protein sample of more than 85% purity as determined by scanning densitometry. The r*Pf*M18AAP sample generally contained low molecular weight contaminants (Figure 1). SDS-polyacrylamide gels indicated that r*Pf*M18AAP migrated at ~67 kDa (Figure 1), which approximates the calculated molecular weight (66.9 kDa) of the recombinant protein.

r*Pf*M18AAP minus the hexahistidine-tag (~65 kDa) separated as three entities with pI 6.6, 6.7 and 6.9 during isoelectric focusing (data not shown). Electrophoresis through non-denaturing gels (Figure 2) and Ferguson plot analysis (Additional file 2) showed that the r*Pf*M18AAP occurred mainly as a monomer, tetramer and higher oligomeric forms. The r*Pf*M18AAP dimer was present in very small amounts. Western blot analysis confirmed that the lower protein band was the r*Pf*M18AAP monomer and that the upper smear included the dimer and tetramer (Figure 2). The smear extended past the tetramer to the top of the gels indicating that there were higher oligomeric forms (e.g. octamer and dodecamer) of r*Pf*M18AAP present. The lower bands seen on the non-denaturing gels were not detected during Western blot analysis with the PentaHis™ HRP Conjugate antibody, indicating that these proteins were contaminants isolated during the purification procedure. Teuscher *et al *showed that recombinant *Pf*M18AAP occurred as an octamer in its active native state by assaying HPLC fractions for enzymatic activity [32]. This is the same number of subunits as the human aspartyl aminopeptidase [27]. However, the unpublished crystal structures of *Clostridium acetobutylicum*, *Thermotoga maritima*, and *Pseudomonas aeruginosa *M18 proteases (Protein Data Bank: ), showed that these enzymes occur as dodecamers. To resolve the issue of the subunit composition of the native *Pf*M18AAP, crystallization of the enzyme will be required.

Enzyme activity assays showed that r*Pf*M18AAP cleaves the N-terminal aspartate from the tripeptide Asp-Ala-Pro-beta-Napthylamide. Teuscher *et al *showed that r*Pf*M18AAP also cleaves dipeptides and that the enzyme is more active on N-terminal glutamates in comparison to N-terminal aspartates [32]. Both these amino acids are acidic indicating that *Pf*M18AAP cleaves peptides and proteins that have an acidic N-terminal amino acid.

**Figure 3:**
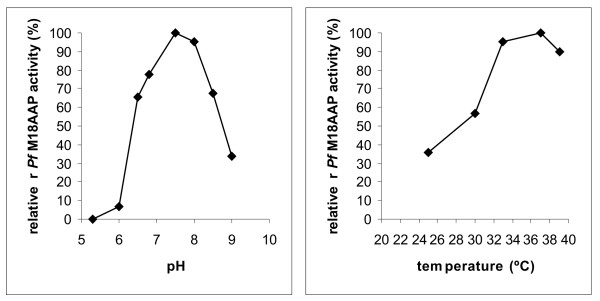
**Relative enzymatic activity of r*Pf*M18AAP over a pH and temperature range**. Graphs showing the relative activity (%) of r*Pf*M18AAP over a pH (left) and temperature (right) range. The pH range included pH values of the parasite food vacuole (pH 5.0–5.4), the parasite cytosol (pH 7.3–7.4) and the erythrocyte cytosol (pH 7.2–7.3). The temperature range was chosen to cover the temperature within the mosquito (26°C) and the human erythrocyte during normal (37°C) and fever (39°C) conditions.

A pH study showed that r*Pf*M18AAP is active from pH 6.8 to 8.5 (Figure 3), with the highest activity at the physiological pH of 7.5 and no great difference in enzyme activity between pH 7.3–7.7. The enzyme had no activity at pH 5.3, which is close to the pH of the food vacuole (5.0–5.2 [34]). The parasite uses the food vacuole for the digestion of hemoglobin into 2–10 amino acid peptides [35,36], which are subsequently exported into the parasite cytosol where they are converted into amino acids by *Pf*M18AAP and other aminopeptidases [32,36]. The parasite cytosol has a pH of 7.3–7.4 [37], allowing *Pf*M18AAP to be active in this parasite compartment. Even though *Pf*M18AAP has no export signals, it has been localized to the parasitophorous vacuole by immunoblot analysis [32] and the erythrocyte membrane by mass spectroscopy [38]. The enzyme could, therefore, also be active in the erythrocyte cytosol which has a neutral pH of 7.2–7.3 [39].

A temperature study revealed that r*Pf*M18AAP is functional from 33–39°C with maximum enzymatic activity at 37°C (Figure 3), which indicates that the enzyme is active when the parasite resides in the human host. r*Pf*M18AAP has 90% enzymatic activity at 39°C (Figure 3), indicating that the enzyme is still functional when the human host experiences fever (maximum temperature 42°C), which occurs after the erythrocytes rupture [40]. *Pf*M18AAP could therefore be active during invasion and growth in new erythrocytes.

### r*Pf*M18AAP binds to spectrin and other erythrocyte membrane skeleton proteins

**Figure 4:**
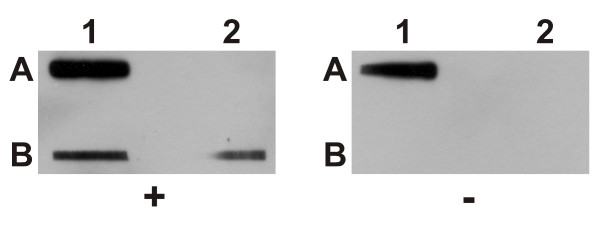
**Blot overlay assay of r*Pf*M18AAP and human erythrocyte spectrin**. Slot blots containing spectrin dimers and tetramers overlaid with r*Pf*M18AAP (+) and Tris buffer (pH 7.5) (-). Slot A1 – r*Pf*M18AAP (positive control); A2 – bovine serum albumin (negative control); B1 – spectrin dimers; B2 – spectrin tetramers. r*Pf*M18AAP was detected with 1:2000 Penta·His™ HRP Conjugate antibody using the SuperSignal^® ^West Pico Chemiluminescent Substrate.

**Figure 5:**
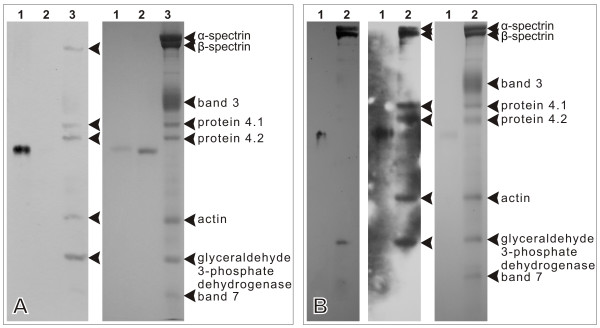
**Blot overlay assay of r*Pf*M18AAP and erythrocyte membrane proteins**. Blot overlay assays were performed four times under hypotonic (A) and isotonic (B) conditions and illustrate the interaction between r*Pf*M18AAP and erythrocyte membrane skeleton proteins. Blot overlays are in the left panels of each figure and the Laemmli SDS-polyacrylamide gels are on the right. **A**. In the absence of salt, and using a short exposure time of 5 minutes for the chemiluminescence reaction, r*Pf*M18AAP binds to beta spectrin, protein 4.1, protein 4.2, actin and G3PD (lane 3 on the immunoblot). Lane 1 – r*Pf*M18AAP (positive control); lane 2 – bovine serum albumin (negative control); lane 3 – erythrocyte membrane proteins. **B**. In the presence of a physiological salt concentration, lane 2 on the blot overlay on the left depicts a short exposure (5 minutes) and shows that r*Pf*M18AAP binds strongly to alpha spectrin, beta spectrin and G3PD. The reaction with beta spectrin is more intense than with alpha spectrin. Lane 2 on the middle blot overlay depicts a longer exposure (20 minutes) and shows that r*Pf*M18AAP also binds to protein 4.1, protein 4.2 and actin. Lane 1 – r*Pf*M18AAP (positive control); lane 2 – erythrocyte membrane proteins.

Blot overlay assays with r*Pf*M18AAP against spectrin (Figure 4) and erythrocyte membrane proteins (Figure 5) confirmed our phage display results, which showed an interaction between spectrin and a 33 amino acid peptide of *Pf*M18AAP [20]. The anti-His6 antibody does not interact with any of the erythrocyte membrane proteins as shown in Figure 1 lane 1 and Additional file 3. Beta spectrin showed much stronger binding to r*Pf*M18AAP than alpha spectrin (Figure 5 and Fairbanks SDS-PAGE, data not shown), which explains why there is no signal evident for alpha spectrin in figure 5A. r*Pf*M18AAP also reacted with other erythrocyte membrane proteins, including protein 4.1, protein 4.2, actin and glyceraldehyde 3-phosphate dehydrogenase (G3PD) (Figure 5). In addition, performing the overlay under isotonic conditions (Figure 5B) increased binding to all the target erythrocyte membrane proteins.

Actin and alpha spectrin could be prime targets for *Pf*M18AAP, because they have three aspartates (actin) and one glutamate (alpha-spectrin) after the N-terminal methionine, which would be cleaved from newly synthesized proteins by the human methionine aminopeptidases [41]. Beta spectrin, protein 4.1 and protein 4.2 contain either an aspartate or a glutamate [32] close to their N-termini. These acidic residues could potentially become available after cleavage of the preceding amino acids by endogenous proteases. Alternatively, another scenario may also be possible. The 33 amino acid binding region of *Pf*M18AAP is on the surface of the enzyme and is not implicated in the catalytic site, which raises the interesting possibility that the enzyme could bind to a protein, but not necessarily cleave it. This could be the case with proteins that do not have an appropriate acidic residue at the N terminus. These proteins could be used as anchors and the enzyme could cleave proteins situated adjacent to it on the membrane, which consists of a network of interacting proteins.

Actin and protein 4.1 are located in the junctional complexes and protein 4.2 is located in the band 3 complex of the erythrocyte membrane [3]. The N-termini of beta spectrin molecules are linked to actin and protein 4.1 in the junctional complex and the N-termini of alpha spectrin are located at the self-association sites of spectrin tetramers [3]. Cleavage of any of these proteins could therefore destabilize and disrupt the junctional complexes, the band 3 complexes and the spectrin tetramers.

### Function of *Pf*M18AAP in the infected erythrocyte

The presumed primary function of *Pf*M18AAP in *P. falciparum *is to cleave aspartates or glutamates from the oligopeptides that are exported from the food vacuole into the parasite cytosol after hemoglobin digestion [32,36]. Amino acids are released from the cell and this regulates the colloid-osmotic pressure within the infected erythrocyte to prevent premature cell lysis and to establish a concentration gradient that facilitates the entry of rare amino acids into the infected erythrocyte [42]. *Pf*M18AAP may therefore be indirectly involved in regulating the volume of the host cell to accommodate the increasing size of the growing parasite.

Our studies, as well as *Pf*M18AAP mRNA and protein data from other laboratories, indicate that the enzyme could also perform additional functions in the parasitophorous vacuole, the erythrocyte cytosol and particularly at the infected erythrocyte membrane skeleton. Microarray data showed that *Pf*M18AAP mRNA is expressed throughout the erythrocytic stages, with the highest expression levels in early and late trophozoites and in gametocytes [43], whilst Northern blot analysis revealed predominant expression in rings [32]. Protein data localized *Pf*M18AAP in merozoites, trophozoites, schizonts [44] and at the infected erythrocyte membrane in trophozoite/schizont stage parasites [38]. Western blot analysis, utilizing anti-*Pf*M18AAP antiserum, also revealed the enzyme in rings, the parasite cytosol and the parasitophorous vacuole [32]. *Pf*M18AAP mRNA is therefore transcribed into the active enzyme at several stages in the erythrocytic life cycle and the *Pf*M18AAP protein is located in several compartments within the parasite-infected erythrocyte. These data imply that *Pf*M18AAP could play a role in parasite invasion, growth and exit from the host cell, since parasite proteins interact with the erythrocyte membrane and the underlying erythrocyte membrane skeleton during intracellular development.

Merozoites are the invasive form of the parasite in erythrocytic schizogony and utilize proteases to gain entry into the new host cell. Inhibition studies with 1,10-phenanthroline have shown that metalloproteases are involved in parasite invasion [45] and hence *Pf*M18AAP could be involved in this process. During invasion and early growth of the parasite, the host experiences febrile paroxysms triggered by the release of toxins during parasitized erythrocyte rupture [40]. Since *Pf*M18AAP is functional at elevated temperatures, we speculate that it may be responsible for regulating, processing and activating other parasite proteins within the ring stage by removing their N-termini.

Several parasite proteases, such as plasmepsin-II [18] and falcipain-2 [19], which are primarily involved in hemoglobin digestion inside the food vacuole, also facilitate the release of the parasite from its erythrocytic host by cleaving membrane skeleton proteins such as spectrin. Studies with inhibitors have proven that parasite proteases first weaken the parasitophorous vacuole membrane and then the erythrocyte membrane prior to parasite release [46], which is caused by an osmotic pressure build-up within the erythrocyte [47]. Weakening of the erythrocyte membrane would involve the disruption of the spectrin-junctional complex network below the plasma membrane. Given that *Pf*M18AAP is located in the parasitophorous vacuole [32] and at the erythrocyte membrane [38], and that the enzyme binds to several erythrocyte membrane proteins and functions at neutral pH, it is, therefore, plausible that *Pf*M18AAP could also play a role in the release of the parasite from its erythrocyte host.

*Pf*M18AAP may represent a novel drug target, since knockdown experiments utilizing a plasmid containing an antisense copy of the PFI1570c gene, resulted in a lethal phenotype [32]. A knockout experiment utilizing a single-crossover strategy [36] produced a truncated PfDAP (*Pf*M18AAP), which retained ~10% enzymatic activity when compared to wild-type parasites. This did not have deleterious effects on parasite replication, possibly because the small amount of active *Pf*M18AAP was sufficient to perform the appropriate enzymatic reactions in the parasite. Additional evidence is thus required to validate *Pf*M18AAP as a drug target. If the enzyme is essential for parasite survival, the development of *Pf*M18AAP inhibitors could lead to new drugs that can be employed to fight malaria infections.

## Conclusion

*Pf*M18AAP binds to erythrocyte spectrin and other erythrocyte membrane proteins. Our evidence and that from other laboratories, suggest that *Pf*M18AAP performs multiple enzymatic functions in the parasite and the erythrocytic host, particularly at the erythrocyte membrane skeleton. These include the final step in hemoglobin digestion, as well as additional roles in erythrocyte invasion, parasite growth and parasite escape from the host cell. These data highlight the multifunctional role of malaria proteases.

## Abbreviations

EBA: erythrocyte binding antigen; FEST: *falciparum*-exported serine/threonine kinase; FIRA: *falciparum *interspersed repeat antigen; G3PD: glyceraldehyde 3-phosphate dehydrogenase; IEF: isoelectric focusing; KAHRP: knob-associated histidine-rich protein; MESA: mature parasite-infected surface antigen; MSP: merozoite surface protein; PfEMP: *P. falciparum *erythrocyte membrane protein; *Pf*M18AAP: *P. falciparum *M18 aspartyl aminopeptidase; RESA: ring-infected erythrocyte surface antigen; SDS-PAGE: sodium dodecylsulphate polyacrylamide gel electrophoresis; SERA: serine-rich antigen

## Competing interests

The authors declare that they have no competing interests.

## Authors' contributions

SBL prepared the recombinant protein, carried out the biochemical characterization, structural analysis, and molecular interaction studies and drafted the manuscript. TLC designed and coordinated the study, and assisted in drafting and editing the manuscript. All authors read and approved the final manuscript.

## Supplementary Material

Additional File 1 **ClustalW alignment of *Pf*M18AAP with *Homo sapiens*, *Saccharomyces cerevisiae*, and other *Plasmodium *homologues**. The sequences, *H. sapiens *(*Hs*) (Q9ULAO), *S. cerevisiae *(*Sc*) (P38821), *P. falciparum *(*Pf*) (PFI1570c), *P. chabaudi chabaudi *(*Pc*) (PC000238.00.0), *P. yoelii yoelii *(*Py*) (PY03205), *P. knowlesi *(*Pk*) (PKH_073050), and *P. vivax *(*Pv*) (Pv087090)) were aligned using the ClustalW program. The five amino acids (blue) that bind the co-factor and the two amino acids (red) that cleave the substrate are conserved amongst all the species. An additional histidine (yellow) involved in enzymatic activity and another histidine (green) involved in quaternary structure stabilization are also marked. The 33 amino acid spectrin-binding region (orange) is only present in the *P. falciparum *aspartyl aminopeptidase. Click here for file

Additional File 2 **Ferguson plot and molecular weight standard curve used to determine the approximate molecular weight of the r*Pf*M18AAP subunits visualized on the non-denaturing agarose/polyacrylamide gels **(figure 2). Ferguson plot (left) showing the log R_m _of the r*Pf*M18AAP oligomeric forms at different polyacrylamide percentages and double-log graph (right) of the negative slopes (obtained from Ferguson plots) versus the molecular weight of each standard (spectrin and BSA multimers). Only three r*Pf*M18AAP subunits (monomer, faint dimer and tetramer) were distinctly visible on the non-denaturing agarose/polyacrylamide gels. The higher oligomers separated as a smear (figure 2). The Ferguson plot (left) shows that r*Pf*M18AAP subunits are oligomers of each other because the lines intersect at ~3% gel concentration. Ferguson plot symbols: squares – r*Pf*M18AAP monomer; triangles – r*Pf*M18AAP dimer; crosses – r*Pf*M18AAP tetramer. The molecular weight of the three r*Pf*M18AAP oligomeric forms was determined from the standard curve (right) as ~70 kDa (monomer), ~155 kDa (dimer), and ~290 kDa (tetramer). Standard curve crosses: yellow – BSA (66 and 132 kDa); blue – spectrin (dimer, 460 kDa; tetramer, 920 kDa; and hexamer, 1380 kDa); red – r*Pf*M18AAP. Click here for file

Additional File 3 **Blot overlay assay performed in the absence of r*Pf*M18AAP**. Laemmli SDS-polyacrylamide gel (left) and blot overlay (right) showing that the PentaHis™ HRP Conjugate antibody does not bind to BSA or any of the erythrocyte membrane proteins when the assay is performed without r*Pf*M18AAP. Lane 1 – bovine serum albumin; lane 2 – r*Pf*M18AAP (positive control); lane 3 – erythrocyte membrane proteins. Click here for file
