# Supplementary material for: The M18 aspartyl aminopeptidase of Plasmodium falciparum binds to human erythrocyte spectrin in vitro
Source: Malar J. 2008 Aug 22;7:161. doi: 10.1186/1475-2875-7-161 (PMC2543045; doi:10.1186/1475-2875-7-161)
Supplement: Additional File 1 — ClustalW alignment of PfM18AAP with Homo sapiens, Saccharomyces cerevisiae, and other Plasmodium homologues. The sequences, H. sapiens (Hs) (Q9ULAO), S. cerevisiae (Sc) (P38821), P. falciparum (Pf) (PFI1570c), P. chabaudi chabaudi (Pc) (PC000238.00.0), P. yoelii yoelii (Py) (PY03205), P. knowlesi (Pk) (PKH_073050), and P. vivax (Pv) (Pv087090)) were aligned using the ClustalW program. The five amino acids (blue) that bind the co-factor and the two amino acids (red) that cleave the substrate are conserved amongst all the species. An additional histidine (yellow) involved in enzymatic activity and another histidine (green) involved in quaternary structure stabilization are also marked. The 33 amino acid spectrin-binding region (orange) is only present in the P. falciparum aspartyl aminopeptidase. [file 1475-2875-7-161-S1.pdf]

```
Hs --MQVAMNGKARKEAVQTAAKELLKFVNRSPSPFHAVAECRNRLLQAGFSELKETEKWN--IKPESKYFMTRNSSTIIAFAVGGQYVPGNG-FSLIGA 93
Sc -MFRIQLRTMSSKTCKSDYPKEFVSFLNSSHSPYHTVHNIKKHLVSNGFKELSERDSWAGHVAQKGKYFVTRNGSSIIAFAVGGKWEPGNP-IAITGA 96
Pf -----------MDKKAREYAQDALKFIQRSGSNFLACKNLKERLENNGFINLSEGETWN--LNKNEGYVLCKENRNICGFFVGKNFNIDTGSILISIG 85
Pc -----------MDKKAREYAQEAIKFIQRSGSSFMACKNLREKLESHGLIHIKEGDQWK--LQKNQGYVLCKENRNICSFFVGKNFNINNGSILISIG 85
Py SFVSVVILFKQMDKKAREYAQEALKFIQRSGSNFMACKNLREKLESHGLIHIKEGDQWK--LQKNQGYVLCKENRNICSFFIGKNFNINNGSILISIG 96
Pk ----MQPTDDTMEKKVRDYAQGAVKFIKKSGSNFLACKNLREKLEEKGFKRIQEGEKWD--LRKNEGYVFSKQNRNICGFFIGKDFNMEKGSILISIG 92
Pv -----------MEKKAREYAQGAVRFIQKSGSNFLACKNLREKLEERGFKRIHEGEKWE--LRKNEGYVLSKQSRNICGFFIGKDFTIEKGSILISIG 85
               .      .:  : *:: * * : :  : :::* . *: .: * : *   :  :  *.. ::. .* .* :* .:   .  : :  .

Hs HTDSPCLRVKRRSRRSQVGFQQVGVETYGGGIWSTWFDRDLTLAGRVIVKCPTSGRLEQQLVHVERPILRIPHLAIHLQRNINENFGPNT--EMHLVP 189
Sc HTDSPALRIKPISKRVSEKYLQVGVETYGGAIWHSWFDKDLGVAGRVFVKDAKTGKSIARLVDLNRPLLKIPTLAIHLDRDVNQKFEFNR--ETQLLP 192
Pf HIDSCALKISPNNNVIKKKIHQINVECYGSGLWHTWFDRSLGLSGQVLYK--KGNKLVEKLIQINKSVLFLPSLAIHLQNRTRYDFSVKINYENHIKP 181
Pc HIDSCALKISPNNKVTKDQISQLNVECYGSGLWHTWFDRSLGLSGQVVYK--KDNKLVEKIIQINKSVLFLPSLAIHLQNRTRYDFSVKVNYENHLKP 181
Py HIDSCTLKISPNNKVTKDQISQLNVECYGSGLWHTWFDRGLGLSGQVVYK--KDNKLIEKIIQINKSVLFLPSLAIHLQNRTRYDFSVKVNYENHLKP 192
Pk HVDTCCLKISPNNNTVKSKVNQLNVECYGSGLWHTWFDRSLGLSGQVVYK--KDDKLVEKLIQINRSIIFLPSLAIHLQNRTRFEFSVKVNFENHLKP 188
Pv HIDSCCLKVSPNNNVVKSKLHQLNVECYGSGLWHTWFDRSLGLSGQVLYK--KEGKLVERLIQINKSLLFLPSLAIHLQNRTRFEFSVKINYEAHLKP 181
   * *:  *::.  ..  .    *:.** **..:* :***:.* ::*:*. *  . .:   :::.:::.:: :* *****:.  . .*  :   * :: *

Hs IL-----ATAIQE-------------------------------------------ELEKGTPEPGPLNAVDER--------------------HHSV 219
Sc IGGLQEDKTEAKT-------------------------------------------EKEINNGEFTSIKTIVQR--------------------HHAE 227
Pf IISTTLFNQLNKCKRNNVHHDTILTTDTKFSHKENSQNKRDDQMCHSFNDKDVSNHNLDKNTIEHLTNQQNEEKNKHTKDNPNSKDIVEHINTDNSYP 279
Pc ILSTVLYEKLIKG-----------------------------------------NENISEKNNS-----STDDEDKNSKN-------------INSSP 220
Py IISTLLYEKLIKG-----------------------------------------NENILEKNISNIDDNNNDDDDMNSKN-------------LNSSP 236
Pk IISTVLYDQLIKGK---------------------------------------EKQNTDAFTEDTLHAEKIQDKCLNGDD--ASPSCLSHQENPNSSP 245
Pv ILSTLLYEHLVKG----------------------------------------GKPGAASPTEDATDADNAQEKRLDAED--HSPSCHSHQENPNSSP 237
   *          :                                                 . .        :                     :

Hs LMSLLCAHLGLS-PKDIVEMELCLADTQPAVLGGAYDEFIFAPRLDNLHSCFCALQALIDSCAGPGSLA----------------------------- 287
Sc LLGLIAKELAIDTIEDIEDFELILYDHNASTLGGFNDEFVFSGRLDNLTSCFTSMHGLT--LAADTEID----------------------------- 294
Pf LLYLLSKELNCK-EEDILDFELCLMDTQEPCFTGVYEEFIEGARFDNLLGSFCVFEGFIELVNSIKNHT-------------SNENTNHTNNITNDIN 363
Pc LLYLLANELKCK-EDDILDFELCLMDTNQPCFTGVYEEFIEGARFDNLLGTFSVFEAYIELIKMIKSE--------------------------NNKN 291
Py LLYLLANELKCK-EEDILDFELCLMDTNKPCFTGVYEEFIEGARFDNLLGTFGVFEAYVELIKNLKNE--------------------------DNEN 307
Pk LLYTLAKELQCE-EKDILDFELCLMDVNEPCFTGAYEEFIEGARFDNLLGSYCVFEAFAEMIDMLKG--------------------KTPPSDGAVLP 322
Pv LLYTLAKELQCQ-EKDILDFELCLMDVNQPCFTGAYEEFIEGARFDNLLGSFCVFEAFAEMVDMLRGGAEAAAGAAAAAEGEASAAGAASAGAAAAPP 334
   *:  :. .*  .  .** ::** * * : . : *  :**: . *:*** . :  :..
```

```
Hs --TEPHVRMVTLYDNEEVGSESAQGAQS-----LLTELVLRRISASCQH--------------PTAFEEAIP----KSFMISADMAHAVHPNYLDKHE 360
Sc --RESGIRLMACFDHEEIGSSSAQGADSNFLPNILERLSILKGDGSDQT--------------KPLFHSAILETSAKSFFLSSDVAHAVHPNYANKYE 376
Pf DNIHNNLYISIGYDHEEIGSLSEVGARSYCTKNFIDRIISSVFKKEIHE-------------KNLSVQEIYGNLVNRSFILNVDMAHCSHPNYPETVQ 448
Pc EPLENNLYICIGYDHEEIGSLSEIGAQSYFTKSFIERIIGNIFKNELKN-------------NETTVDEIYGSLSSRSLILNVDMAHCGHPNYPETIQ 376
Py --LGNNLYICIGYDHEEIGSLSEIGAQSYFTKNFIERILGNIFKNELKN-------------NDITIDEIYGSLSNRSLILNVDMAHCGHPNYPETIQ 390
Pk PEAHANLYICIGYDHEEIGSLSEVGAQSYFTQNFIKRILTAISSSQVGQNTHP--------SSPTSIDELYGSLMSRSLILNVDMAHCSHPNYPETVQ 412
Pv PGAHANLYICIGYDHEEIGSLSEVGAQSYFTQNFIKRILAAVCSSHACDAASATTSSAATASAPPSIDELYGSLMSRSLLLNVDMAHCSHPNYPETVQ 432
         : :   :*:**:** *  ** *     :: .:     .                      ...       :*:::. *:**. **** :. :

Hs ENHRPLFHKGPVIKVNSKQRYASNAVSEALIR------EVANKVKVPLQDLMVRNDTPCGTTIGPILASRLGLRVLDLGSPQLAMHSIREMACTTGVL 452
Sc SQHKPLLGGGPVIKINANQRYMTNSPGLVLVK------RLAEAAKVPLQLFVVANDSPCGSTIGPILASKTGIRTLDLGNPVLSMHSIRETGGSADLE 468
Pf DNHQLFFHEGIAIKYNTNKNYVTSPLHASLIKRTFELYYNKYKQQIKYQNFMVKNDTPCGSTVGSMVAANLSMPGIDIGIPQLAMHSIREIAAVHDVF 546
Pc QSHHLRFHDGIAIKYNTNKNYVTSPYYACLLKRTFELYQNQYNQKIKYQNFMIKNDTPCGSTVGSMVASNLSMPGMDIGISQLAMHSIREIAAIHDIY 474
Py QNHHLRFHEGIAIKYNTNKNYVTSPYYACLLKRTFELYQNQNNQKIKYQNFMIKNDTPCGSTVGSMVAANLSMPGMDIGIPQLAMHSIRELAAIHDIY 488
Pk ANHQLFFHEGIAIKYNTNRNYATSPYYTCLLKRTFELFTSKFNEKIKYQNFMVKNDTPCGSTVGSMVASNLSMPGVDIGIPQLAMHSIREIAAVRDIY 510
Pv ASHQLFFHEGIAIKYNTNKNYVTSPYYTCLLKRTFELFASNFNEKIKYQNFMVKNDTPCGSTVGSMVAANLSMPGIDIGIPQLAMHSIREIAAVRDVY 530
    .*:  :  * .** *:::.* :..    *::            ::  * ::: **:***:*:*.::*:. .:  :*:* . *:****** .   .:

Hs QTLTLFKGFFELFPSLSHNLLVD- 475
Sc FQIKLFKEFFERYTSIESEIVV-- 490
Pf FLIKGVFAFYTYYNQVLSTCVHDK 570
Pc YLIKGVFAFYAYYNQVLSTCVHDA 498
Py YLVKGIFAFYAYYNQVLSSCVHDS 512
Pk YLVKGILAFYTYYSHVHASCVPDE 534
Pv YLVKGVLAFYAYYSHVLASCVPDA 554
     :. .  *:  :  :    :
```
