# Supplementary figures and images for: The M18 aspartyl aminopeptidase of Plasmodium falciparum binds to human erythrocyte spectrin in vitro
Source: Malar J. 2008 Aug 22;7:161. doi: 10.1186/1475-2875-7-161 (PMC2543045; doi:10.1186/1475-2875-7-161)

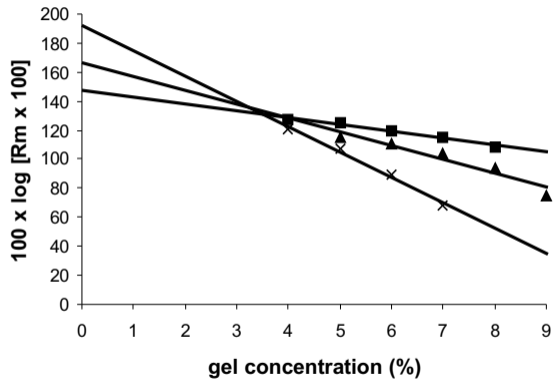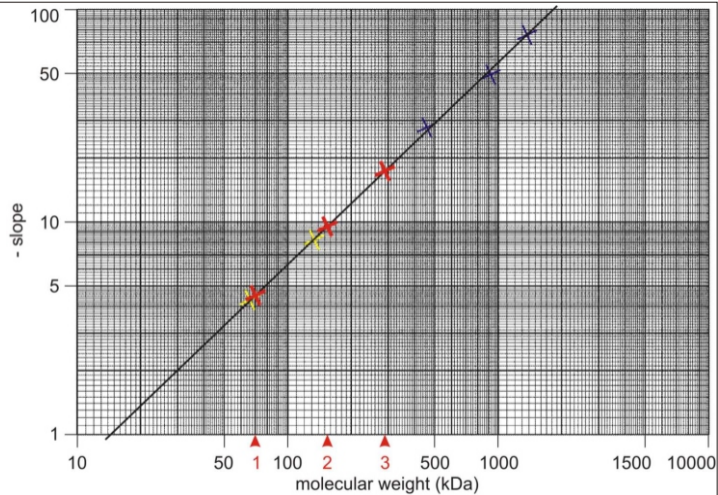

Supplement: Additional File 2 — Ferguson plot and molecular weight standard curve used to determine the approximate molecular weight of the rPfM18AAP subunits visualized on the non-denaturing agarose/polyacrylamide gels (figure 2). Ferguson plot (left) showing the log Rm of the rPfM18AAP oligomeric forms at different polyacrylamide percentages and double-log graph (right) of the negative slopes (obtained from Ferguson plots) versus the molecular weight of each standard (spectrin and BSA multimers). Only three rPfM18AAP subunits (monomer, faint dimer and tetramer) were distinctly visible on the non-denaturing agarose/polyacrylamide gels. The higher oligomers separated as a smear (figure 2). The Ferguson plot (left) shows that rPfM18AAP subunits are oligomers of each other because the lines intersect at ~3% gel concentration. Ferguson plot symbols: squares – rPfM18AAP monomer; triangles – rPfM18AAP dimer; crosses – rPfM18AAP tetramer. The molecular weight of the three rPfM18AAP oligomeric forms was determined from the standard curve (right) as ~70 kDa (monomer), ~155 kDa (dimer), and ~290 kDa (tetramer). Standard curve crosses: yellow – BSA (66 and 132 kDa); blue – spectrin (dimer, 460 kDa; tetramer, 920 kDa; and hexamer, 1380 kDa); red – rPfM18AAP. [file 1475-2875-7-161-S2.pdf]
