# Supplementary material for: The M18 aspartyl aminopeptidase of Plasmodium falciparum binds to human erythrocyte spectrin in vitro
Source: Malar J. 2008 Aug 22;7:161. doi: 10.1186/1475-2875-7-161 (PMC2543045; doi:10.1186/1475-2875-7-161)
Supplement: Additional File 3 — Blot overlay assay performed in the absence of rPfM18AAP. Laemmli SDS-polyacrylamide gel (left) and blot overlay (right) showing that the PentaHis™ HRP Conjugate antibody does not bind to BSA or any of the erythrocyte membrane proteins when the assay is performed without rPfM18AAP. Lane 1 – bovine serum albumin; lane 2 – rPfM18AAP (positive control); lane 3 – erythrocyte membrane proteins. [file 1475-2875-7-161-S3.pdf]

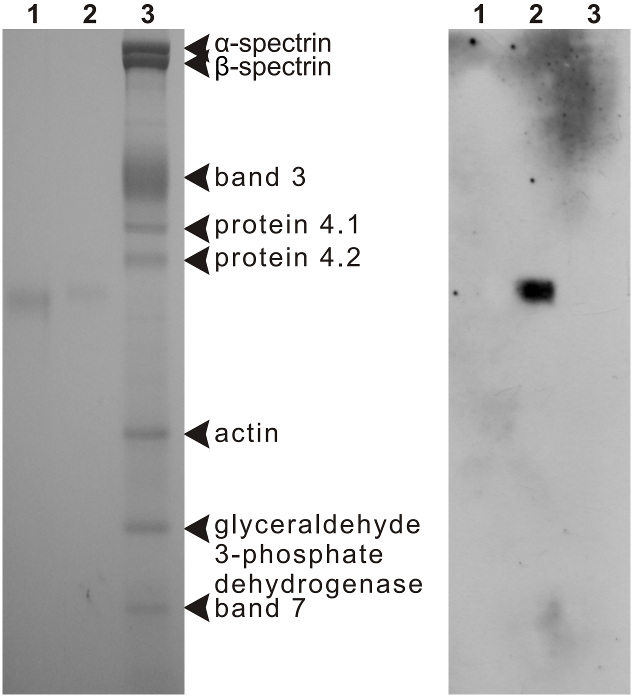
1
2
3
α-spectrin
β-spectrin
band 3
protein 4.1
protein 4.2
actin
glyceraldehyde
3-phosphate
dehydrogenase
band 7
1
2
3
